# Effect of carotid sinus massage in terminating the episodes of supraventricular tachycardia

**DOI:** 10.1097/MD.0000000000045199

**Published:** 2025-10-17

**Authors:** Muhammad Shaheer Bin Faheem, Wajeeha Imam, Hafiza Qurat Ul Ain, Faheem Feroze, Amina Saliha, Aasma Ismail, Syed Tawassul Hassan, Muhammad Idrees Khan

**Affiliations:** a Department of Internal Medicine, Karachi Institute of Medical Sciences, KIMS, Karachi, Pakistan; b CMH Multan Institute of Medical Sciences, Multan, Pakistan; c Combined Military Hospital Rawalpindi (CMH), Rawalpindi, Pakistan; d Karachi Medical and Dental College, KMDC, Karachi, Pakistan; e Adam University School of Medicine, Bishkek, Kyrgyzstan.

**Keywords:** arrhythmia, carotid sinus massage, efficacy, supraventricular tachycardia, tachyarrhythmia

## Abstract

Supraventricular tachycardia (SVT) is a common cardiac arrhythmia marked by an abnormally fast heart rate, leading to significant patient discomfort and frequent visits to emergency departments. Carotid sinus massage (CSM) is a widely used vagal maneuver employed to rapidly stop SVT episodes. This research investigates the success and safety profile of CSM in treating SVT among Pakistani patients, offering critical insights for its use in emergency medical practices. A prospective observational cohort study was carried out in the Medicine Department of CMH Hospital, Multan, Pakistan, from January 1, 2024 to June 30, 2024. The study included 200 SVT patients aged 18 and older who presented at the emergency department. Information on patient demographics, medical history, and current clinical status was documented. Patients were laid supine with little neck extension. Gentle pressure was applied to the carotid sinus for 5 to 10 seconds on alternating sides, with a 1-minute interval if needed. The effectiveness of the procedure was determined by the rate of SVT termination, defined as a return to normal sinus rhythm within 30 seconds. Safety was monitored by noting any adverse events during and after the procedure. The average age of the study participants was 55.55 ± 8.86 years. Females made up 59.5% of the sample. CSM successfully terminated SVT in 18.5% of the cases. Adverse effects that were reported included dizziness (3.5%), vomiting (2.5%), syncope (1.5%), exacerbation of arrhythmia (3.5%), and temporary neurological issues (1%). This study assessed the effectiveness and safety of CSM in managing SVT. The findings indicate an 18.5% success rate in SVT termination, consistent with previous studies. Despite moderate efficacy and some side effects, CSM remains a valuable non-pharmacological approach for SVT management, with certain contraindications to be considered.

## 1. Introduction

Supraventricular tachycardia (SVT) is a specific type of tachycardia that involves atrial tissue up to the atrioventricular junction with rates >100 bpm.^[1]^ It has 2 predominant subtypes that are atrioventricular nodal reentrant tachycardia and atrioventricular reentrant tachycardia.^[2]^ These arrhythmias are estimated to impact 2.25 out of every 1000 people in the general population across the world, and their incidence rises with advancing age, with prevalence being double in females than males of all age groups.^[3]^ However, epidemiological data on tachyarrhythmias is limited in low- and middle-income countries.^[4]^

SVT patients may experience severe discomfort and anxiety, which may result in recurrent visits to the emergency departments and, in certain situations, hospitalization.^[2,5]^ The management of SVT has evolved over the years, including both acute termination strategies and long-term prevention approaches. Vagal maneuvers (VMs) are considered the initial therapeutic intervention for SVT for hemodynamically stable patients, aiming to decelerate or potentially terminate the arrhythmia.^[2,6]^ Among the VMs, CSM is a readily available and often employed technique that has been used for decades. CSM stimulates the carotid baroreceptor directly by applying gentle pressure on the bifurcation of the common carotid artery for 5 seconds.^[7,8]^ This technique reflexively lowers sympathetic activity and raises parasympathetic tone by stimulating carotid body baroreceptors, which cause a reduction in blood pressure and heart rate. CSM breaks the reentrant circuit, causing SVT and restores the normal sinus rhythm.^[9]^ However, CSM has been utilized in clinical practice since the beginning of the 20th century, and its application in identifying and managing a range of cardiac conditions has gradually improved.

The efficacy of CSM in terminating SVT depends on several factors such as massage style, age, kind and duration of SVT, and underlying cardiac condition. For instance, atrioventricular reentrant tachycardia and atrioventricular nodal reentrant tachycardia respond more readily to CSM than other types of SVT. Despite the long-term use of CSM, its safety and efficacy remained an area of debate in medical research, and its effectiveness has been questioned when compared to other VMs or pharmacological approaches in spite of studies showing its success rates ranging from 20 % to 50 %.^[10,11]^

The tolerability of CSM is another crucial component that requires close consideration. While many individuals manage the procedure with ease, some people may have anxiety or uneasiness, or they may experience transient side effects, including lightheadedness or dizziness during or immediately after the massage. Furthermore, the clinical value of CSM as a first therapeutic strategy for managing SVT may be significantly influenced by the patient’s subjective experience with the technique and their receptivity to further interventions in recurring episodes. In geriatric groups, CSM should be approached with caution. Furthermore, due to the possibility of carotid atheroembolism and cerebrovascular events, patients with significant carotid artery disease or a history of cerebrovascular episodes are likely to be at a higher risk, even in the absence of an auscultated bruit. Therefore, measuring the incidence and severity of these adverse reactions is essential for directing treatment strategies and promoting discussion with patients before the procedure. Therefore, rigorous pre-procedure assessment, adequate patient selection, and appropriate techniques are necessary to reduce the likelihood of adverse outcomes.^[11,12]^

Pakistan’s young demographic may have an impact on SVT rates as well since some forms are more common in younger people than others. As previously mentioned, incidence varies throughout communities, making it challenging to determine without specific studies.^[13]^ SVT is still a major clinical problem in Pakistan, even though it is not considered extremely common. This study aimed to ascertain whether CSM had any influence on the cessation of SVT episodes in our local patients. This study will provide helpful data for using CSM in emergency setups in clinical practice.

## 2. Methods

This prospective observational cohort study was conducted at the Department of Medicine, CMH Hospital Multan, from the 1st of January 2024 to the 30th of June 2024 over a period of 6 months.

The sample size was calculated as per the following assumptions:

Precision = 4.00%, prevalence = 9.10%.^[14]^

Ceylan et al (2019) reported a prevalence rate of 9.10%, which was utilized to estimate sample size. The initial success rate for CSM in terminating SVT was also documented as 9.1%. This provides a useful benchmark for establishing the appropriate sample size with reasonable precision and confidence.

Population size = infinite, with 95% confidence interval.

Estimated sample size: n = 199.

Two hundred patients of any gender, aged above 18 years and presenting to the emergency department with SVT (confirmed by 12-lead electrocardiography [ECG]), were enrolled in this study through consecutive sampling.

All clinicians performing CSM were trained using a standardized protocol approved by the department’s senior cardiology staff. All the patients underwent auscultation for carotid bruits prior to the procedure, and those with detected bruits or have a history of carotid artery disease, stroke, transient ischemic attack, myocardial infarction (within the last 3 months), uncontrolled hemodynamics, severe hypotension, or those taking medications that can affect heart rate were excluded from the study.

Patients’ demographics, medical history and current clinical characteristics (including vital signs like heart rate, blood pressure, and oxygen saturation) were recorded.

Patients were placed in a supine position with the neck slightly extended. The carotid sinus was located at the level of the thyroid cartilage, and gentle pressure was applied for 5 to 10 seconds, alternating between the right and left sides, if necessary, at an interval of 1 minute. Heart rate and rhythm were continuously monitored using ECG during and up to 5 minutes after the procedure.

Post-procedure ECG and all the relevant clinical details were also recorded in the given format. All the procedures were done by trained clinicians following standard protocols.

The effect of the procedure was assessed by the success rate of SVT termination (the return to sinus rhythm within 30 seconds of the procedure).

Safety was measured by any incidence of adverse events (dizziness, nausea, syncope, exacerbation of arrhythmia or any change in vital signs, hypotension, or neurological symptoms).

Approval for conducting the study was obtained from the hospital’s ethical committee.

Prior consent was taken from patients/guardians before enrollment in the study.

Data was analyzed using SPSS version 25. Quantitative variables were expressed in mean ± SD, while qualitative variables were expressed in frequency and percentage. Descriptive analysis was used to summarize patient characteristics and study outcomes. Logistic regression analysis was performed to predict the success of treatment.

## 3. Results

The analysis covered a total of 200 patients. The average age was 55.55 ± 8.86 years (range: 43–72), with 59.5% being female. The majority of patients (39%) arrived at the emergency department within 2 hours after the SVT episode. The average systolic and diastolic blood pressures were 115.93 ± 9.84 and 78.42 ± 5.54 mm Hg, respectively. At admission, the average pulse rate was 78.42 ± 5.54 beats/min, with SO_2_ of 96.51 ± 1.74%. The reported pulse rate represents vital indicators taken at the time of admission, not during the active SVT episode. Many patients had spontaneously resolved or received prehospital therapies (such as vagal movements or medication), leading to a return to sinus rhythm. It was done before their vitals were recorded. As a result, the mean pulse rate reflects the heart rate after the arrhythmia has resolved rather than during active SVT. Detailed demographic and clinical data are presented in Table 1.

**Table 1:** Demographics, medical history, and clinical findings, n = 200.

Demographics	
Age (mean ± SD), years	55.55 ± 8.86
Gender	
Male, n (%)	81 (40.5)
Female, n (%)	119 (59.5)
Patient clinical findings at the time of admission	
Patients reporting within 2 h after the episode, n (%)	78 (39)
Systolic BP (mean ± SD) mm Hg	115.93 ± 9.84
Diastolic BP (mean ± SD) mm Hg	78.42 ± 5.54
Pulse (beat/min)	78.42 ± 5.54
SO_2_ (%)	96.51 ± 1.74
Temperature (°C)	36.55 ± 0.23
Medical history	
Previous history of SVT, n (%)	86 (43)
CAD,* n (%)	64 (32)
Hypertension, n (%)	59 (29.5)
Diabetes, n (%)	38 (19)
Anemia, n (%)	27 (13.5)
Hyperthyroidism, n (%)	9 (4.5)

SVT = supraventricular tachycardia.

* Coronary artery disease.

**Figure 1.**
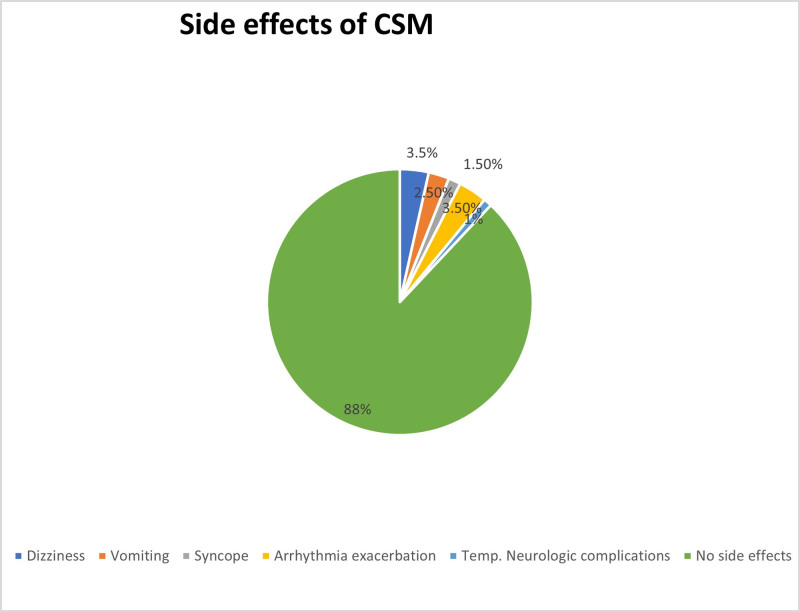
Safety profile.

CSM was effective in terminating SVT in 18.5% of patients (n = 37). This shows that CSM might be an effective first non-pharmacologic intervention for certain individuals. Table 2 shows the outcomes connected to CSM effectiveness. In terms of procedural safety, 88% of patients reported no adverse effects, showing an acceptable and appropriate safety profile. Figure 1 illustrates the distribution of adverse effects.

**Table 2:** Effect of CSM on SVT, n = 200.

Effect of the procedure		
Termination of SVT, n (%)	Yes, n (%)	37 (18.5)
	No, n (%)	163 (81.5)

CSM = carotid sinus massage, SVT = supraventricular tachycardia.

To distinguish between responders and nonresponders, baseline variables were evaluated in patients with and without effective SVT cessation. Patients in the CSM success group had a substantially lower mean age than nonresponders (52.4 ± 7.2 vs 56.7 ± 9.1 years, *P* = .04), suggesting that younger persons may benefit more from CSM. However, no statistically significant differences were seen for hypertension (24% vs 31%, *P* = .41) or a history of SVT (49% vs 41%, *P* = .29). These findings are detailed in Table 3.

**Table 3:** Comparison of baseline characteristics between patients with and without successful SVT termination using CSM.

Variable	CSM success (n = 37)	No success (n = 163)	*P*-value (chi-square or *t* test)
Mean age	52.4 ± 7.2	56.7 ± 9.1	.04
HTN (yes, %)	24%	31%	.41
Previous SVT (yes, %)	49%	41%	.29

CSM = carotid sinus massage, HTN = hypertension, SVT = supraventricular tachycardia.

Binary logistic regression was used to determine whether clinical or demographic characteristics might predict the effectiveness of CSM. The variables analyzed were age, gender, systolic and diastolic blood pressures, pulse rate, temperature, SO_2_, and comorbidities such as hypertension, diabetes, and coronary artery disease. None of the variables were statistically significant (*P* > .05), but systolic blood pressure exhibited a tendency toward significance (B = 0.035, *P* = .132), indicating a probable but unconfirmed relationship with CSM response. Table 4 displays the complete regression findings.

**Table 4:** Binary logistic regression predicting successful termination of SVT by CSM.

Variable	B (coefficient)	SE	Wald	*P*-value
Age	0.020	0.021	0.886	.347
Gender (M/F)	‐0.120	0.395	0.092	.762
HTN	‐0.147	0.478	0.094	.759
Previous history of SVT	‐0.439	0.382	1.316	.251
Patient reporting < 2 h	‐0.164	0.400	0.168	.682
SBP	0.035	0.023	2.267	.132
DBP	‐0.013	0.040	0.110	1
SO_2_	‐0.192	0.119	2.616	1
Temperature (°C)	‐0.365	0.381	0.918	1
Pulse rate (beats/min)	0.014	0.013	1.240	1
Hyperthyroidism	‐0.695	0.897	0.600	1
Anemia	‐0.533	0.517	1.064	1
Diabetes	‐0.104	0.489	0.045	1
CAD	‐0.115	0.414	0.077	1

CAD = coronary artery disease, CSM = carotid sinus massage, DBP = diastolic blood pressure, HTN = hypertension, SBP = systolic blood pressure, SO2 = oxygen saturation, SVT = supraventricular tachycardia.

**Figure 2.**
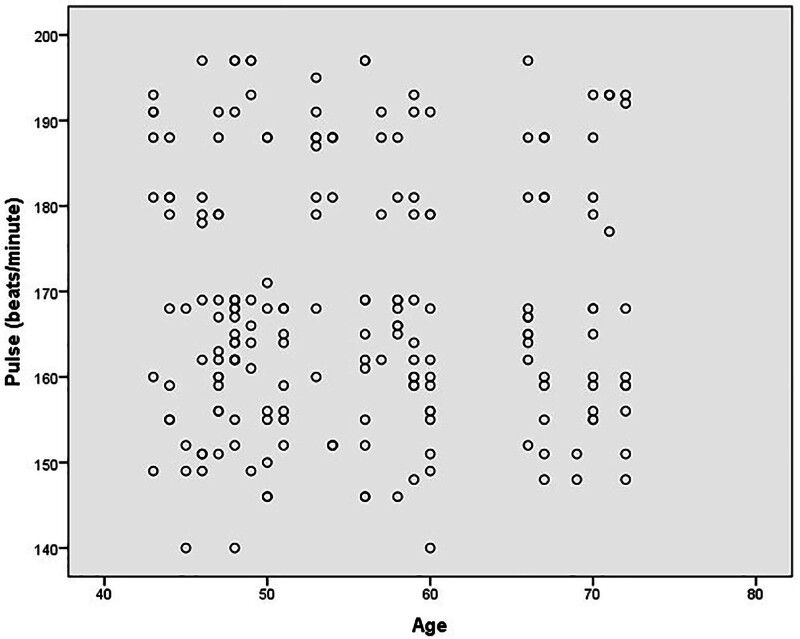
Scatter plot of pulse rate by participant age.

To visually explore the relationship between age and pulse rate, a scatter plot was generated, as shown in Figure 2. A clear linear pattern was absent, which reinforces the point that pulse rates recorded post-presentation may not accurately reflect SVT episode severity or duration.

## 4. Discussion

Our study aimed to assess the immediate safety and efficacy of CSM in resolving the episodes of SVT in the Pakistani cohort. This is done with continuous monitoring of ECG for almost up to 5 minutes following the procedure, and the findings demonstrated a success rate of 18.5% with a low incidence of adverse events such as dizziness, syncope and transient neurological complications. We contributed to the literature by adding new regional data on the safety and efficacy of CSM in an understudied population of South Asia. However, studies suggest that the stimulation of the vagus nerve and cholinergic pathways can reduce the atrial refractory period without altering conduction velocity.^[15]^ CSM is a noninvasive VM that stimulates the vagus nerve, leading to enhanced cholinergic activity, which slows down AV nodal conduction and restores normal sinus rhythm by terminating reentrant causing SVT. It is highly effective in patients with unexplained syncope and SVT, primarily nondiabetic individuals and those treated within 2 hours of SVT onset, with success rates ranging from 10% to 20%.^[8,16]^ Moreover, it is reported to have a 14% success rate after failed VSM.^[8]^

Several studies have compared the efficacy of CSM with different VM techniques, such as the standard Valsalva maneuver and modified Valsalva maneuver (MVasM). A randomized control trial conducted by Ceylan E demonstrated comparable findings between CSM and standard Valsalva maneuver with the absence of statistical significance after 1- and 5-minute periods of the procedure. However, MVasM proved to be superior among all approaches, with a success rate of 43.7%, while achieving statistical significance at both time points and reporting fewer side effects.^[14]^ In contrast, the efficacy of CSM in our study was relatively low, which can be attributed to factors including operator-dependent variability and patient level differences like age, neck anatomy, autonomic tone and delayed presentation at the emergency department as only 39% of the patients presented within 2 hours of symptom onset which may significantly reduce responsiveness of CSM underscoring the need for improved emergency staff training and CSM standardization. The efficacy of MVasM was further strengthened by a meta-analysis of 14 RCTs, indicating no significant difference between CSM and SVaSM while representing the dominance of MVasM in reducing SVT episodes.^[17]^ Appelboam et al showed that MVasM outperformed standard VM techniques while providing the idea that CSM should be followed or combined with CSM.^[18]^ Our findings support this strategy while emphasizing that CSM can still serve as a first-line approach, especially when time and resources are limited.

Furthermore, our study displayed a favorable safety profile of CSM beyond its uncertain success rate as recorded adverse events were temporary and self-limiting, aligning with the existing literature, which shows higher tolerance and resolution of adverse events such as hypotension, nausea, dyspnea, and dizziness upon CSM termination.^[17]^ Nevertheless, contraindications such as carotid artery disease, prior transient ischemic attack/stroke, or the presence of carotid bruits can increase the risk of complications during CSM and must be assessed strictly before the procedure.^[19]^ Additionally, we addressed a significant gap by analyzing and providing such data from Pakistan, where research and training related to SVT management are limited and factors like genetic predisposition, disparities in healthcare access and lifestyle patterns, including high caffeine intake and sedentary behavior can influence both the incidence and response to SVT treatments.

Arrhythmias, including SVT, atrial fibrillation (often due to rheumatic heart disease), and bradycardia, are ranked third among cardiovascular issues after coronary artery disease and heart failure, and it is important to understand SVT within the broader category of arrhythmia.^[13]^ SVT can act as a trigger for atrial fibrillation, particularly among patients with preexisting structural heart disease.^[20]^ Further, certain rare forms of SVT, including those associated with channelopathies or neuromuscular disorders such as Friedreich Ataxia, which may not respond to VMs, highlight the importance of clinician awareness in recognizing and managing these rare cases.^[21]^ However, the main limitation of this study is its observational methodology and short follow-up period following the operation. Therefore, remission beyond the acute phase was not investigated. Future studies covering these limitations will add to this valuable data for patients suffering from SVT.

## 5. Conclusions

The findings of this research support the notion that with CSM, a moderate percentage of patients have reduced heart rate and alleviated SVT symptoms. CSM is a safe and well-tolerated intervention for managing SVT episodes and offers an easy-to-use, non-pharmacological approach to addressing SVT-related discomfort.

## Acknowledgments

The services of paramedic staff for taking and maintaining patient record is acknowledged.

## Author contributions

**Conceptualization:** Hafiza Qurat Ul Ain, Wajeeha Imam.

**Data curation:** Muhammad Shaheer Bin Faheem, Wajeeha Imam, Syed Tawassul Hassan.

**Formal analysis:** Muhammad Shaheer Bin Faheem.

**Methodology:** Muhammad Shaheer Bin Faheem.

**Project administration:** Muhammad Shaheer Bin Faheem, Syed Tawassul Hassan, Muhammad Idrees Khan.

**Resources:** Faheem Feroze, Amina Saliha, Aasma Ismail, Syed Tawassul Hassan.

**Software:** Muhammad Shaheer Bin Faheem, Faheem Feroze, Amina Saliha, Aasma Ismail.

**Supervision:** Muhammad Shaheer Bin Faheem.

**Validation:** Muhammad Shaheer Bin Faheem, Wajeeha Imam, Hafiza Qurat Ul Ain.

**Visualization:** Hafiza Qurat Ul Ain, Faheem Feroze, Amina Saliha, Aasma Ismail.

**Writing – original draft:** Muhammad Shaheer Bin Faheem, Wajeeha Imam, Hafiza Qurat Ul Ain, Syed Tawassul Hassan.

**Writing – review & editing:** Muhammad Shaheer Bin Faheem, Syed Tawassul Hassan, Faheem Feroze.
